# PVminerLLM: structured extraction of patient voice from patient-generated text using large language models

**DOI:** 10.1038/s44482-026-00039-1

**Published:** 2026-10-27

**Authors:** Samah Jamal Fodeh, Linhai Ma, Ganesh Puthiaraju, Srivani Talakokkul, Afshan Khan, Ashley Hagaman, Sarah Lowe, Aimee Roundtree

**Affiliations:** 1 Yale University, New Haven, CT USA; 2 Texas State University, San Marcos, TX USA

**Keywords:** Computational biology and bioinformatics, Health care, Mathematics and computing, Medical research

## Abstract

Patient-generated text contains critical information about patients’ lived experiences, social circumstances, and engagement in care, including factors that strongly influence adherence, care coordination, and health equity. However, these patient voice signals are rarely available in structured form, limiting their use in patient-centered outcomes research and clinical quality improvement. Reliable extraction of such information is therefore essential for understanding and addressing non-clinical drivers of health outcomes at scale. We introduce PVminer, a benchmark for structured extraction of patient voice, and propose PVminerLLM, a supervised fine-tuned large language model tailored to this task. Across multiple datasets and model sizes, PVminerLLM substantially outperforms prompt-based baselines, achieving up to 84.18% F1 for Code prediction, 81.39% F1 for Sub-code prediction, and 88.24% F1 for evidence Span extraction. Notably, strong performance is achieved even with smaller models, demonstrating that reliable patient voice extraction is feasible without extreme model scale. These results enable scalable analysis of social and experiential signals embedded in patient-generated text.

## Introduction

Patient-generated data, including secure messages, survey responses, and interview narratives, provide a rare and direct window into patients’ lived experiences outside traditional clinical encounters^1–4^. Unlike structured clinical records, these texts capture how individuals articulate their needs, constraints, emotions, and expectations in their own words. Collectively, such expressions constitute the patient voice, a socially embedded signal that reflects not only clinical concerns but also broader social, environmental, and relational contexts that shape health outcomes. As used throughout this work, the term “patient voice” refers exclusively to these written, textual expressions and is distinct from voice-based or acoustic signals used in speech analysis and Voice AI applications. These contexts include social determinants of health, such as housing instability and financial insecurity, as well as patients’ engagement, preferences, and participation in managing their care^5–10^.

Despite its importance, the patient voice remains largely underexplored in patient-centered outcomes research and health services research. When patient-generated text is analyzed, it is often reduced to narrow, isolated categories or surface-level entities, obscuring the complex and overlapping social realities embedded in everyday language. For example, a single patient message can simultaneously convey medical uncertainty, emotional distress, social constraints, and preferences regarding care decisions. Yet most computational approaches fail to preserve this richness, instead focusing on a limited subset of social factors^11–23^. As a result, critical social signals that influence adherence, care coordination, partnership and shared decision-making, and equity are systematically underrepresented in large-scale analyses^24–26^.

For conditions such as mental health and substance use disorders, where much of care occurs outside traditional clinical settings and depends heavily on sustained patient engagement, outcomes hinge not only on access to therapy or medication but also on patients’ ability to manage stigma, social stressors, and fluctuating motivation in daily life. Evidence shows that treatment adherence and continuity of care in depression, anxiety, and opioid use disorder are strongly influenced by psychosocial factors, such as housing, food, financial insecurity, stress, and perceived social support^27,28^. Scalable methods that can reliably extract these signals from unstructured text are therefore essential for understanding treatment effectiveness and for designing responsive interventions to patients’ needs. However, manual abstraction of the patient voice from textual data and its transformation into a structured, accessible format is laborious and expensive^11,29–35^. Existing machine learning methods^36–46^ offer scalability but have primarily focused on clinical notes in electronic health records rather than unstructured patient-generated text, and typically target only a limited set of social domains.

In this study, we introduce the PVminer framework, which formalizes the patient voice annotation as schema-constrained structured prediction from unstructured patient-generated text. The task requires extracting hierarchical labels with the main domains including: partnership and building rapport; shared decision-making; socioemotional support; and social determinants of health, and associated evidence Spans. PVminer reflects intrinsic properties of patient-generated data, including highly unstructured language, a wide range of social factors, overlapping categories, severe label imbalance, and a small number of semantically critical tokens that determine annotation correctness.

To provide an initial solution using state-of-the-art scalable techniques, we first implement a dedicated prompt engineering approach and benchmark a range of instruction-tuned large language models spanning model sizes from 1.5B to 70B parameters under zero-shot and few-shot settings. Carefully engineered prompts enable these models to capture coarse semantic signals and yield measurable performance improvements. However, they frequently produce poorly structured, verbose, or truncated outputs. This behavior leads to substantial precision-recall gaps, indicating that prompting alone is insufficient for reliable patient voice extraction and motivating task-specific model adaptation.

To address these limitations, we introduce PVminerLLM, a suite of supervised fine-tuned language models specialized for the PVminer task. By adapting instruction-tuned models to the PVminer schema, PVminerLLM enforces structured, schema-valid outputs and achieves strong performance across hierarchical labels and evidence Spans. Our results demonstrate that supervised fine-tuning offers a scalable and effective approach for high-fidelity extraction of socially and clinically meaningful signals from patient-generated text, enabling downstream analysis of the patient voice at scale. The contributions of this work are as follows.

- We propose the PVminer Framework, a structured prediction formulation for extracting patient voice from patient-generated text that captures hierarchical labels and evidence Spans. To the best of our knowledge, this is the first benchmark for structured extraction of relational, socioemotional, and social determinant signals from patient-generated secure messages.
- We propose a systematically engineered prompt that integrates six prompting principles: chain-of-thought decomposition, single-turn self-validation, schema contracting, direction-aware control logic, expert-derived disambiguation rules, and role priming, into a unified instruction framework for token-critical structured generation. Under zero-shot conditions, this engineered prompt substantially improves Code, Sub-code, and Span extraction across all evaluated models compared to a baseline prompt, and serves as the instruction template for supervised fine-tuning of PVminerLLM.
- We develop PVminerLLM, a suite of supervised fine-tuned language models specialized for the PVminer task, providing a practical and effective solution for patient voice extraction across diverse healthcare settings.
- Through systematic evaluation across model sizes, label frequencies, data sources, and evaluation thresholds, we demonstrate that PVminerLLM achieves strong extraction performance, reduces Span hallucination, and maintains comparable performance from 1.5B to 70B parameters, establishing practical readiness for real-world deployment.

## Results

We evaluate the PVminer benchmark across prompt-based and supervised fine-tuning settings, systematically comparing extraction performance across model sizes, label types, data sources, and evaluation thresholds. Evaluation metrics and experimental setup are described in the “Methods” section.

### Baseline and engineered prompt performance on the PVminer benchmark

Table 1 reports F1 scores for Code, Sub-code, and Span extraction under a zero-shot setting. In both the baseline and engineered prompt configurations, no in-context examples (i.e., no 1-shot or few-shot demonstrations) were provided to the model. The two settings differ solely in the design of the instruction. The baseline prompt consists of a minimal task description with general guidance, whereas the engineered prompt instruction introduces structured output formatting, explicit decision logic, disambiguation rules, self-validation constraints, and performance-oriented guidance. This comparison isolates the effect of our proposed prompt engineering method (section “Prompt engineering”) without altering the number of shots.

**Table 1:** Comparison of F1 scores (%) between Baseline and Engineered prompts under zero-shot conditions, reported as mean ± std over 3 runs

Model	Method	Code	Sub-code	Span
Llama3.3-70B	Baseline	56.92 ± 0.56	25.23 ± 0.43	46.19 ± 0.44
	Engineered	**62.65** ± 0.66	**44.79** ± 0.66	**55.19** ± 0.56
Llama3.1-8B	Baseline	0.00 ± 0	0.00 ± 0	49.24 ± 1.85
	Engineered	**50.47** ± 2.00	**23.66** ± 1.27	**52.82** ± 1.17
Llama3.2-3B	Baseline	22.96 ± 0.40	6.45 ± 0.21	25.91 ± 1.25
	Engineered	**38.54** ± 0.46	**10.95** ± 0.66	**33.58** ± 1.90
Qwen2.5-1.5B	Baseline	13.23 ± 0.26	1.95 ± 0.61	16.25 ± 0.73
	Engineered	**19.93** ± 0.14	**3.92** ± 0.77	**22.40** ± 1.13

The baseline and engineered prompt variants are provided in Supplementary Information. Our proposed *engineered prompt* will serve as the instruction template for the SFT. For each model and task in this table, the higher F1 between the baseline and engineered prompt is shown in bold.

Across all four models, the engineered prompt instruction consistently achieves higher F1 scores across all prediction targets. In particular, prompt engineering yields substantial gains in Code and Sub-code extraction for the 8B model and produces clear improvements across all tasks for the 70B model, with similar gains also observed for the 3B and 1.5B models. These findings demonstrate that explicit structural guidance significantly enhances the reliability of multi-label, structured extraction in the PVminer benchmark, even under a strictly zero-shot regime.

In addition to the overall F1-score improvements, from experimental observations, predictions on several commonly confused Codes and Sub-codes decreased as our proposed engineered prompt is introduced. For example, at the Code level, major swaps such as PartnershipPatient predicted as PartnershipProvider decrease from 18 to 5 cases, and the reverse direction decreases from 12 to 4 cases, reflecting clearer role distinction and more accurate label assignment overall. At the Sub-code level, large cross-confusions such as Salutation predicted as Signoff decrease from 24 cases to 7 cases, and Signoff predicted as Salutation decreases from 11 to 3 cases, reflecting better separation between opening and closing message components.

### Engineered prompt and supervised fine-tuning performance

Table 2 reports the proposed engineered prompt zero-shot and few-shot performance on the PVminer benchmark across instruction-tuned models of varying sizes. For zero-shot, while larger models achieve moderate overall F1 scores, performance remains limited across all settings, particularly for Sub-code prediction and Span recall. Even for the largest model, recall for fine-grained labels is substantially lower than precision, reflecting difficulty in producing complete and schema-valid structured outputs under zero-shot prompting. These results indicate that zero-shot prompting alone is insufficient for reliable extraction on the PVminer task. One-shot and Two-shot provide incremental improvements. Besides the overall performance increasing, as the number of shots grows, the difference between precision and recall is decreasing. But one-shot and two-shot results still exhibit similar failure patterns. Recall is still significantly lower than precision, even for the largest model, which motivates supervised fine-tuning.

**Table 2:** Code-, sub-code-, and span-level performance of the engineered prompt (0/1/2-shot) and supervised fine-tuning (SFT)

Model	Method	Metric	Code	Sub-code	Span
Qwen2.5-1.5B	0-shot	P	33.30 ± 0.72	21.30 ± 4.73	65.75 ± 5.71
		R	14.23 ± 0.11	2.16 ± 0.42	13.50 ± 0.61
		F1	19.93 ± 0.14	3.92 ± 0.77	22.40 ± 1.13
	1-shot	P	44.10 ± 2.92	29.84 ± 1.15	75.99 ± 5.32
		R	23.63 ± 1.64	8.28 ± 0.60	25.55 ± 0.85
		F1	30.77 ± 2.04	12.96 ± 0.82	38.22 ± 1.42
	2-shot	P	50.35 ± 1.98	31.93 ± 2.43	61.98 ± 3.64
		R	31.58 ± 1.03	11.47 ± 1.35	40.42 ± 1.79
		F1	38.82 ± 1.34	16.86 ± 1.69	48.92 ± 2.23
	SFT	P	**84.08** ± **0.05**	**75.00** ± **0.00**	**82.25** ± **0.57**
		R	**70.34** ± **0.31**	**65.85** ± **0.23**	**83.02** ± **0.08**
		F1	**76.60** ± **0.17**	**70.13** ± **0.13**	**82.63** ± **0.32**
Llama3.2-3B	0-shot	P	43.09 ± 0.94	15.74 ± 0.52	49.93 ± 4.08
		R	34.90 ± 1.41	8.41 ± 0.78	25.32 ± 1.42
		F1	38.54 ± 0.46	10.95 ± 0.66	33.58 ± 1.90
	1-shot	P	57.43 ± 1.09	31.50 ± 0.38	66.37 ± 3.21
		R	36.53 ± 1.96	13.80 ± 0.40	26.71 ± 0.78
		F1	44.64 ± 1.64	19.19 ± 0.31	38.09 ± 1.09
	2-shot	P	60.00 ± 4.06	35.36 ± 1.32	71.12 ± 0.07
		R	41.53 ± 3.15	18.80 ± 1.85	36.54 ± 1.72
		F1	49.08 ± 3.56	24.53 ± 1.83	48.26 ± 1.50
	SFT	P	**81.83** ± **0.40**	**74.59** ± **0.44**	**84.34** ± **0.40**
		R	**77.64** ± **0.53**	**75.21** ± **0.73**	**86.19** ± **0.10**
		F1	**79.68** ± **0.45**	**74.90** ± **0.57**	**85.25** ± **0.26**
Llama3.1-8B	0-shot	P	49.30 ± 1.60	33.01 ± 0.50	75.23 ± 1.47
		R	51.72 ± 2.53	18.46 ± 1.38	40.73 ± 1.85
		F1	50.47 ± 2.00	23.66 ± 1.27	52.82 ± 1.17
	1-shot	P	54.43 ± 1.61	41.70 ± 1.23	72.52 ± 1.50
		R	55.15 ± 0.18	31.87 ± 1.30	56.00 ± 1.10
		F1	54.78 ± 0.90	36.13 ± 1.29	63.18 ± 0.26
	2-shot	P	61.51 ± 0.96	45.41 ± 2.01	74.61 ± 0.76
		R	59.98 ± 0.46	36.83 ± 2.19	62.56 ± 1.42
		F1	60.73 ± 0.32	40.67 ± 2.14	68.04 ± 0.52
	SFT	P	**85.29** ± **0.42**	**79.27** ± **1.13**	**85.55** ± **0.30**
		R	**78.30** ± **1.55**	**78.78** ± **0.79**	**88.08 ±** **0.29**
		F1	**81.64** ± **1.02**	**79.02** ± **0.94**	**86.79** ± **0.29**
Llama3.3-70B	0-shot	P	70.05 ± 1.11	52.03 ± 1.02	84.16 ± 0.17
		R	56.66 ± 0.37	39.33 ± 0.45	41.06 ± 0.61
		F1	62.65 ± 0.66	44.79 ± 0.66	55.19 ± 0.56
	1-shot	P	71.68 ± 1.09	51.10 ± 1.24	86.76 ± 0.85
		R	62.08 ± 1.28	46.78 ± 1.38	53.99 ± 0.50
		F1	66.54 ± 1.12	48.85 ± 1.32	66.56 ± 0.45
	2-shot	P	72.40 ± 1.16	54.64 ± 0.53	**88.49 ±** **0.56**
		R	62.27 ± 0.73	49.29 ± 1.31	56.39 ± 0.66
		F1	66.95 ± 0.85	51.82 ± 0.73	68.88 ± 0.47
	SFT	P	**87.26** ± **0.36**	**82.57** ± **0.32**	87.81 ± 0.37
		R	**81.32** ± **0.58**	**80.25** ± **0.27**	**88.68** ± **0.23**
		F1	**84.18** ± **0.21**	**81.39** ± **0.20**	**88.24** ± **0.29**

Values are in %, reported as mean ± std over 3 runs. For each model and task, the best P, R, and F1 values across methods are highlighted in bold.

Table 2 also reports performance after 10 epochs of supervised fine-tuning across models of varying sizes. Llama3.3-70B-Instruct consistently outperforms the other models, achieving F1 scores of 84.18% for Code classification, 81.39% for Sub-code classification, and 88.24% for Span extraction. Notably, the performance of the medium and smaller models is comparable to the Llama3.3-70B-Instruct model, allowing flexibility in model selection as needed. Compared to zero- and few-shot prompting, supervised fine-tuning yields substantially higher precision, recall, and F1-score across all tasks, with particularly strong gains for Code and Sub-code prediction. Compared to zero-shot learning, Llama3.3-70B-Instruct achieves relative F1 improvements of 34.37% (Code), 81.71% (Sub-code), and 59.88% (Span). Relative to 2-shot learning, the corresponding improvements are 25.74, 57.06, and 28.11%, respectively.

### Span hallucination analysis

To assess the extent of Span hallucination, we compute the percentage of predicted Spans that appear as exact substrings of the input message (Table 3). Across all model sizes, SFT models achieve very high grounding rates, ranging from 98.68 to 99.27%, corresponding to hallucination rates of at most 1.32%. In contrast, two-shot models show consistently higher hallucination rates: 1.87% for 70B, 3.58% for 8B, 7.03% for 3B, and 10.16% for 1.5B. Qualitatively, two-shot hallucinations include substantively fabricated phrases absent from the input, whereas SFT outputs more reliable evidence from the source message.

**Table 3:** Hallucination rate (%, mean ± std over 3 runs), defined as the percentage of predicted spans that are not exact substrings of the input message

Model	SFT	Two-shot
Qwen2.5-1.5B	1.32 ± 0.18	10.16 ± 2.39
Llama3.2-3B	1.31 ± 0.40	7.03 ± 0.97
Llama3.1-8B	0.88 ± 0.19	3.58 ± 0.11
Llama3.3-70B	0.73 ± 0.06	1.87 ± 0.17

### Performance stratified by data source

Performance stratification by data source (Table 4) shows that PVminerLLM generalizes across institutional settings and that this finding holds consistently across all four model sizes. Notably, TXACC messages from charitable clinics, which contribute greater linguistic and socioeconomic diversity, achieve the highest Sub-code F1 across all four models (ranging from 75.55% for Qwen2.5-1.5B to 89.67% for Llama3.3-70B), suggesting that the models are not biased toward the academic medical center (YNHH) data that constitutes the majority of instances. In fact, YNHH generally yields the lowest Code F1 under SFT despite its larger share of data. Survey responses consistently show the lowest Sub-code and Span F1 across all models, reflecting stylistic differences between free-text survey responses and structured secure messages.

**Table 4:** F1 of all four models at the Code, Sub-code, and Span levels, stratified by data source (micro-averaged, in %, mean ± std over 3 runs)

Model	Setting	Survey	TXACC	YNHH
*Code F1*				
Qwen2.5-1.5B	Two-shot	26.63 ± 2.99	34.70 ± 5.29	41.03 ± 0.74
	SFT	**75.44** ± **0.20**	**81.88** ± **0.49**	**75.43** ± **0.12**
Llama3.2-3B	Two-shot	55.43 ± 13.90	52.93 ± 4.79	47.49 ± 2.95
	SFT	**87.90** ± **1.21**	**82.16** ± **0.33**	**78.21** ± **0.55**
Llama3.1-8B	Two-shot	73.65 ± 3.92	62.98 ± 1.67	58.86 ± 0.43
	SFT	**87.14** ± **0.62**	**86.20** ± **1.35**	**79.94** ± **1.07**
Llama3.3-70B	Two-shot	77.18 ± 2.24	69.55 ± 0.80	65.27 ± 0.83
	SFT	**88.89** ± **0.00**	**91.28** ± **0.00**	**81.95** ± **0.29**
*Sub-code F1*				
Qwen2.5-1.5B	Two-shot	16.08 ± 5.32	15.02 ± 3.29	17.34 ± 1.58
	SFT	**54.88** ± **0.58**	**75.55** ± **1.81**	**69.51** ± **0.27**
Llama3.2-3B	Two-shot	15.38 ± 15.38	25.91 ± 1.99	24.62 ± 1.28
	SFT	**71.57** ± **1.70**	**81.16** ± **0.13**	**73.52** ± **0.79**
Llama3.1-8B	Two-shot	46.04 ± 1.08	41.48 ± 5.87	40.15 ± 1.87
	SFT	**65.17** ± **1.69**	**82.98** ± **1.15**	**78.71** ± **0.87**
Llama3.3-70B	Two-shot	54.07 ± 1.28	50.88 ± 4.42	51.96 ± 0.24
	SFT	**67.03** ± **0.62**	**89.67** ± **0.23**	**80.06** ± **0.19**
*Span F1*				
Qwen2.5-1.5B	Two-shot	47.47 ± 2.83	44.03 ± 4.15	50.49 ± 2.37
	SFT	**71.38** ± **1.42**	**84.57** ± **0.39**	**83.06** ± **0.62**
Llama3.2-3B	Two-shot	41.69 ± 4.05	46.18 ± 2.53	49.46 ± 1.37
	SFT	**71.20** ± **0.34**	**85.05** ± **0.21**	**86.55** ± **0.37**
Llama3.1-8B	Two-shot	65.20 ± 2.76	60.58 ± 2.62	70.42 ± 0.16
	SFT	**80.45** ± **0.22**	**86.42** ± **1.04**	**87.46** ± **0.10**
Llama3.3-70B	Two-shot	66.23 ± 2.59	65.59 ± 1.74	70.04 ± 0.34
	SFT	**80.62** ± **0.60**	**89.30** ± **0.56**	**88.63** ± **0.30**

For each model and data source, the higher F1 between the two-shot and SFT settings is shown in bold.

### Patient voice domains identified using a two-shot engineered prompt and PVminerLLM

Within the engineered prompt, the two-shot setting yielded the highest performance, so we analyze the domains identified based on two-shot as representative performance and compare them with PVminerLLM results. From Tables 5 and 6, in the two-shot setting, performance differs substantially across patient voice domains and reflects their prevalence in the dataset (Fig. 2). PartnershipPatient and PartnershipProvider are among the most prevalent domains and achieve the highest performance, with F1 scores of 84.43 and 87.84%, respectively. These domains capture frequent patterns of collaboration and involvement in patient–provider exchanges, which are often explicitly expressed in secure messages. Their strong performance suggests that two-shot prompting can reliably identify structurally clear patient voice signals. SDOH-related content is also prevalent, particularly sub-codes such as HealthCareAccessAndQuality and EconomicStability. For the SDOH code, the Two-shot engineered prompt achieves an F1 of 57.94%, with high precision (80.21%) but lower recall (45.36%), indicating that socioeconomic concerns are accurately detected when predicted but remain under-identified overall. CareCoordinationProvider, another relatively common domain, shows moderate performance (F1 = 63.31%), whereas CareCoordinationPatient performs less consistently (F1 = 42.79%). In contrast, SharedDecisionPatient and SharedDecisionProvider are less frequent in the corpus and exhibit substantially lower performance (F1 = 46.25 and 35.61%, respectively). At the Sub-code level, rare or nuanced categories such as alignment (F1 = 12.08%), checkingUnderstanding/clarification (F1 = 12.35%), and inviteCollaboration (F1 = 29.38%) show low recall and reduced overall performance, and several infrequent sub-codes collapse to 0.00%. These findings indicate that two-shot prompting is more effective for prevalent and linguistically explicit patient voice domains but struggles to reliably identify low-frequency or context-dependent behaviors. Overall, domain-level variation in performance aligns with distributional imbalance, underscoring the need for task-specific model adaptation to improve detection of less common patient voice signals.

**Table 5:** Two-shot and SFT performance of 70B model at the Code level (micro-averaged per class, in %, mean ± std over 3 runs)

	Two-shot			SFT		
Code	P	R	F1	P	R	F1
CareCoordinationPatient	49.72	37.61	42.79 ± 4.39	81.81	69.23	**74.98** ± **1.74**
CareCoordinationProvider	56.88	71.43	63.31 ± 5.31	83.20	80.00	**81.54** ± **1.18**
PartnershipPatient	88.30	80.89	84.43 ± 0.49	91.11	86.01	**88.49** ± **0.40**
PartnershipProvider	91.32	84.62	87.84 ± 0.58	92.94	86.81	**89.77** ± **0.00**
SDOH	80.21	45.36	57.94 ± 0.88	91.71	87.70	**89.66** ± **0.66**
SharedDecisionPatient	40.91	53.33	46.25 ± 2.36	79.20	76.19	**77.66** ± **1.03**
SharedDecisionProvider	40.13	32.05	35.61 ± 5.45	51.35	38.46	**43.87** ± **2.29**
SocioEmotionalBehaviour	57.93	47.85	52.37 ± 0.96	83.53	79.03	**81.22** ± **0.39**

For each Code, the higher F1 between the two-shot and SFT settings are shown in bold.

**Table 6:** Two-shot and SFT performance of 70B model at the Sub-code level (micro-averaged per class, in %, mean ± std over 3 runs), organized by parent Code and sorted by test-set prevalence within each group

		Two-shot			SFT		
Sub-code	Prev.	P	R	F1	P	R	F1
*Partnership (Patient and Provider, combined)*							
Clinical Care	18.0	75.76	43.45	54.96 ± 3.26	72.31	83.93	**77.69** ± **0.00**
salutation	11.1	93.12	92.44	92.77 ± 1.78	100.00	98.97	**99.48** ± **0.00**
signoff	9.7	67.50	73.33	70.29 ± 2.49	98.44	98.43	**98.43** ± **0.34**
Appreciation/Gratitude	3.5	48.22	82.80	60.92 ± 2.24	100.00	90.32	**94.92** ± **0.00**
connection	3.0	25.89	30.77	27.81 ± 6.83	75.27	82.05	**78.49** ± **2.61**
alignment	2.7	47.22	6.94	12.08 ± 10.97	95.65	91.67	**93.62** ± **0.00**
build trust	0.9	0.00	0.00	0.00 ± 0.00	57.87	62.50	**60.05** ± **2.12**
*PartnershipPatient only*							
activeParticipation/involvement	10.4	79.10	44.69	57.07 ± 5.18	85.50	82.05	**83.74** ± **0.56**
expressOpinions	4.3	35.39	85.09	49.99 ± 2.04	81.08	78.95	**80.00** ± **0.00**
statePreferences	1.6	32.51	52.38	40.05 ± 8.73	64.44	69.05	**66.67** ± **3.98**
*PartnershipProvider only*							
inviteCollaboration	3.4	55.89	20.00	29.38 ± 7.58	61.94	72.22	**66.68** ± **3.68**
maintainCommunication	2.4	44.20	44.44	44.17 ± 7.03	73.54	69.84	**71.55** ± **0.21**
checkingUnderstanding/clarification	1.6	12.82	11.90	12.35 ± 11.32	67.78	45.24	**54.23** ± **3.56**
requestsForOpinion	1.3	29.26	42.42	34.57 ± 3.85	55.71	30.30	**39.08** ± **4.78**
*SDOH*							
HealthCareAccessAndQuality	7.3	42.95	23.96	30.74 ± 2.53	82.21	69.79	**75.49** ± **0.86**
EconomicStability	4.5	84.40	17.95	29.51 ± 3.29	87.75	79.49	**83.41** ± **0.64**
SocialAndCommunityContext	3.2	58.98	61.90	60.38 ± 5.93	79.00	94.05	**85.86** ± **1.07**
NeighborhoodAndBuiltEnvironment	2.5	55.56	27.27	36.28 ± 4.36	66.08	59.09	**62.36** ± **3.42**
EducationAccessAndQuality	0.5	100.00	25.00	40.00 ± 0.00	100.00	83.33	**90.48** ± **8.25**
*SharedDecisionPatient*							
SeekingApproval	2.7	42.62	33.33	37.07 ± 9.31	83.33	83.33	**83.33** ± **0.00**
ExploreOptions	1.4	12.91	30.56	18.11 ± 1.27	56.67	47.22	**51.52** ± **5.25**
ApprovalofDecision/Reinforcement	0.2	16.67	33.33	21.67 ± 20.21	33.33	50.00	**40.00** ± **0.00**
*SharedDecisionProvider*							
MakeDecision	2.3	52.69	26.67	35.36 ± 8.96	60.73	35.00	**44.28** ± **2.17**
ShareOptions	0.9	20.06	37.50	26.12 ± 1.14	48.15	50.00	**49.02** ± **1.70**
Approval/Reinforcement	0.5	0.00	0.00	0.00 ± 0.00	21.67	25.00	**23.15** ± **1.60**

Sub-codes shared between PartnershipPatient and PartnershipProvider are combined. Codes without Sub-codes (SocioEmotionalBehaviour, CareCoordinationPatient, CareCoordinationProvider) are excluded; their performance is reported in Table 5. For each Sub-code, the higher F1 between the two-shot and SFT settings are shown in bold.

Prev. (%) denotes the percentage of test-set Sub-code annotations belonging to each Sub-code.

In contrast to the two-shot setting, PVminerLLM substantially improves identification across nearly all patient voice domains, particularly those that are both prevalent and clinically meaningful. At the code level, the most prevalent domains, PartnershipPatient and PartnershipProvider, reach F1 scores of 88.49 and 89.77%, respectively, improving from 84.43% and 87.84% under two-shot prompting. Similarly, the SDOH domain improves markedly from 57.94 to 89.66% F1, with recall increasing substantially, indicating that socioeconomic concerns are no longer systematically under-identified. CareCoordinationPatient and CareCoordinationProvider also show meaningful gains (42.79 to 74.98% and 63.31 to 81.54%, respectively), reflecting stronger detection of logistical and treatment-related content. Notably, SharedDecisionPatient improves from 46.25 to 77.66% F1, and SharedDecisionProvider from 35.61 to 43.87%, suggesting that supervised fine-tuning better captures explicit decision-making behaviors that were previously difficult to identify with few-shot prompting. At the Sub-code level, improvements are especially significant for prevalent and clinically important signals. Clinical Care increases from 54.96 to 77.69%, EconomicStability from 29.51 to 83.41%, and SocialAndCommunityContext from 60.38 to 85.86%. High-frequency structural markers such as salutation and signoff remain strong (99.48% and 98.43% F1 under PVminerLLM), while several relational Sub-Codes that previously showed low recall under two-shot prompting demonstrate substantial gains, including activeParticipation/involvement (57.07–83.74%) and connection (27.81–78.49%). Although a small number of extremely rare Sub-Codes remain at or near zero performance, the overall pattern indicates that supervised fine-tuning reduces the performance gap between prevalent and less frequent patient voice domains.

### Qualitative case analysis

To provide interpretable evidence of how PVminerLLM resolves failure modes observed under prompting, we present three case analyses comparing two-shot and SFT predictions on representative test-set messages (full details in Supplementary Information). In Case 1, a dense patient message containing six Code–Sub-code pairs across clinical, SDOH, and socioemotional domains, two-shot extracts only two correct pairs, confusing the Sub-code *activeParticipation/involvement* with *expressOpinions* and merging distinct SDOH and socioemotional signals; PVminerLLM identifies all six. In Case 2, two-shot assigns exclusively Provider-side Codes to a patient-authored message, achieving zero correct pairs due to direction confusion; PVminerLLM correctly identifies Patient-side Codes. In Case 3, two-shot misses all *NeighborhoodAndBuiltEnvironment* signals related to weather and transportation barriers, misclassifying a transportation concern as *expressOpinions* and confusing *signoff* with *Appreciation/Gratitude*; PVminerLLM captures all three SDOH references with correct Sub-codes.

## Discussion

This study systematically examined the ability of instruction-tuned large language models to perform schema-constrained structured extraction on the PVminer task under prompt-based and supervised adaptation settings. Our experiments indicate that prompt-based inference alone cannot reliably achieve structured extraction under the PVminer constrained annotation schema, highlighting the need for additional mechanisms or fine-tuning. Even with carefully designed prompts, both zero-shot and few-shot settings show large gaps in recall, especially for Sub-code prediction and evidence Span extraction. Many errors come from outputs that do not fully follow the required structure, are incomplete, or mix up closely related labels. These findings suggest that while instruction-tuned models can often understand the general meaning of a message, they struggle to consistently produce fully structured outputs without task-specific supervision.

Supervised fine-tuning, on the other hand, leads to clear and consistent improvements across all parts of the task. The large gaps between precision and recall observed in zero-shot and few-shot performance (Table 2) are largely reduced after fine-tuning, indicating more complete and schema-consistent structured outputs. Span extraction achieves consistently high F1 scores across all models, and performance differences across model scales are reduced, supporting our claim that task-specific supervision enables reliable structured extraction on the PVminer task without reliance on extreme model size. This indicates that the main challenge of PVminer is not just understanding the text itself, but also learning how to reliably translate patient-generated language into structured, Span-based annotations under strict formatting rules. Compared to few-shot prompting, which can only identify highly frequent and structurally explicit domains, supervised fine-tuning enables more consistent and balanced detection across both prevalent and nuanced patient voice behaviors. This finding is particularly important for domains such as SDOH and shared decision-making, which carry significant implications for understanding patient needs and support. Furthermore, the effect of model size becomes much smaller after fine-tuning. While larger models still perform slightly better, smaller models reach comparable performance once adapted to the task. This suggests that successful patient voice extraction depends more on task alignment and high-quality supervision than on model size alone. As a result, accurate extraction can be achieved with smaller models of the PVminerLLM’s suite of supervised fine-tuned LLMs, making large-scale and practical deployment more feasible in healthcare settings.

Notably, label frequency and linguistic difficulty are confounded in the PVminer schema. The Sub-codes with the lowest performance across both two-shot and SFT settings are also among the rarest and most pragmatically ambiguous. Approval/Reinforcement (provider) reaches 0% under two-shot and remains low under SFT (23.15%), build trust reaches 0% under two-shot and 60.05% under SFT, and ApprovalofDecision/Reinforcement (patient) exhibits highly unstable two-shot performance (21.67 ± 20.21%). These Sub-codes share a common linguistic property: they require recognizing implicit communicative intent rather than explicit lexical cues. For example, provider reinforcement of a decision is rarely expressed through a formulaic phrase; it is conveyed through brief validating cues that could equally be classified as alignment or connection depending on context.

The ability to accurately extract the patient voice domains from patient-generated text has important implications for patient-centered care and health equity. Many signals that matter for clinical outcomes are social rather than purely medical^1–7^. Patients often describe emotional stress, difficulty paying for care, transportation problems, caregiving responsibilities, or uncertainty about treatment decisions in their own words. These experiences shape whether patients can follow treatment plans, stay engaged with care teams, and feel supported, yet they are rarely captured in structured clinical records and are therefore easy to overlook in routine care. By focusing on unstructured patient-generated text, our proposed PVminerLLM defines and captures Codes such as Social And Community Context, Neighborhood and Built Environment, Socioeconomic Status helps make these social and lived experiences visible at scale. Instead of relying on manual chart review or small qualitative studies, health systems can systematically extract and summarize patient voice signals across large populations. This makes it possible to identify patterns that are difficult to see otherwise, such as common barriers to adherence, frequent sources of confusion or distress, or groups of patients who may need additional support beyond standard clinical care.

In clinical practice, these capabilities can support more informed and responsive care. Structured patient voice information can help care teams recognize when patients are struggling with social or emotional challenges, even if those issues are not explicitly raised during visits. It can also guide targeted interventions, such as referrals to social services, adjustments to care plans, or additional follow-up for patients at risk of disengagement. By better aligning care with patients’ real-world circumstances, health systems can improve both effectiveness and patient experience.

Importantly, our results show that scalable patient voice extraction does not require extremely large models or highly specialized data. This makes the approach more accessible to a wide range of healthcare settings, including community clinics and resource-constrained systems. From a research perspective, PVminerLLM provides essential infrastructure for incorporating patient voice into patient-centered outcomes research. By enabling large-scale analysis of social context and lived experience alongside clinical information, this work supports more complete assessments of care quality, equity, and effectiveness, and helps ensure that patients’ voices are meaningfully represented in data-driven healthcare decisions.

The proposed PVminerLLM framework demonstrates strong capability in extracting and structuring patient voice content, effectively mapping patient-generated language to clinically and socially meaningful domains. However, we plan to extend this work to enhance its practical and clinical deployment and reduce its complexity. First, the current prompt design is necessarily large and complex due to the hierarchical schema, strict output constraints, and need for disambiguation rules. A promising next step is the use of multi-agent or modular inference frameworks, where distinct agents handle complementary subtasks such as semantic interpretation, label selection, and Span verification. Decomposing the task in this way may reduce prompt complexity while improving robustness and interpretability^47–51^. Second, while supervised fine-tuning yields strong performance, post-SFT alignment remains underexplored for structured extraction tasks like PVminer. Preference-based or constraint-aware alignment methods tailored to token-critical outputs could further improve reliability, particularly for rare Sub-codes and boundary-sensitive Spans^52–57^. We aim to further strengthen the reliability, scalability, robustness, and clinical utility of patient voice extraction systems, moving closer to practical deployment in real-world healthcare settings.

In this work, we introduced the PVminer task and benchmark for structured extraction of patient voice from patient-generated text. By formalizing patient voice annotation as a schema-constrained task with hierarchical labels and Span grounding, we provided a rigorous testbed for evaluating large language models under realistic clinical constraints. Our benchmark results show that prompt-based inference alone is insufficient for reliable extraction, while supervised fine-tuning yields substantial and consistent improvements across models of varying sizes.

The proposed PVminerLLM models demonstrate that accurate and scalable patient voice extraction can be achieved without reliance on extreme model scale, making deployment more feasible in real-world healthcare settings. By enabling systematic measurement of social and experiential signals embedded in patient-generated text, PVminer supports more holistic patient-centered research and lays the groundwork for integrating the patient voice into data-driven clinical and health services applications.

## Methods

This section describes the PVminer task formulation, dataset construction and annotation, the prompt engineering and supervised fine-tuning approaches, and the evaluation methodology used throughout this study.

### PVminer task formulation

We define the PVminer task as a schema-constrained structured extraction problem over patient-generated text (i.e., portal messages). Given a single message, the task requires identifying all relevant patient voice expressions and representing each as a structured output consisting of a Code, a Sub-code, and a grounding text Span. This formulation is designed to support systematic evaluation of large language models under realistic constraints, where outputs must be both semantically correct and strictly schema-valid. Unlike conventional single-label or flat multi-label classification settings, PVminer allows multiple structured outputs to be extracted from a single message. Each output corresponds to a distinct expression that may reflect concerns, social context, or lived experience. The inclusion of Span grounding further requires models to localize evidence precisely within the input text, rather than relying on coarse semantic matching.

The labeling schema is organized hierarchically. Codes represent high-level semantic categories, while sub-codes capture more granular distinctions. A single Code may be associated with multiple Sub-codes, and certain Sub-codes may be shared across different Codes. This structure reflects the overlapping and non-exclusive nature of patient-generated language and introduces additional constraints that models must enforce during extraction. Definitions of all Codes and Sub-codes are provided in Supplementary Information. The empirical distribution of each Code and Sub-code is shown in Fig. 2.

Formally, let *s* denote a patient-generated message and let *d* ∈ {*Y*, *N*} be a message-direction indicator, where *Y* denotes provider-to-patient messages, and *N* denotes patient-to-provider messages. Let ${\mathcal{C}}=\{{c}_{1},\ldots ,{c}_{8}\}$ denote the set of Codes and ${\mathcal{U}}=\{{u}_{1},\ldots ,{u}_{25}\}$ denote the set of Sub-codes. A hierarchical constraint mapping ${\mathcal{M}}:{\mathcal{C}}\to {2}^{{\mathcal{U}}}$ specifies the set of valid Sub-codes associated with each Code.

Given an input message, the extraction model *g*_*ϕ*_ produces a set of structured outputs:

1 $${g}_{\phi }(s,d)\ =\ {\mathcal{E}}(s)$$

2 $${\mathcal{E}}(s)\subseteq {\mathcal{C}}\times {\mathcal{U}}\times {\mathcal{R}}(s)$$

where ${\mathcal{R}}(s)$ denotes the set of all contiguous text Spans within *s*. Each extracted element $(c,u,r)\in {\mathcal{E}}(s)$ consists of a Code $c\in {\mathcal{C}}$, a Sub-code $u\in {\mathcal{M}}(c)$, and an evidence Span $r\in {\mathcal{R}}(s)$ that grounds the extracted label in the original text.

A single message may yield one or multiple such tuples, reflecting the variable density, overlap, and compositional nature of patient voice expressions in the text.

This task formulation serves as the foundation for benchmarking large language models under zero-shot and few-shot prompting, as well as for supervised fine-tuning to enforce schema adherence and improve structured extraction quality. In the following sections, we describe dataset construction and annotation, prompt-based extraction strategies, supervised fine-tuning for PVminer, and evaluation protocols tailored to structured, Span-grounded outputs.

### Datasets and annotation

Our study uses a corpus of patient-generated text (i.e., messages and surveys) collected from multiple healthcare and research settings, including secure messaging from Yale New Haven Health, electronic messages from charitable clinics affiliated with the Texas Association for Charitable Clinics, and free-text patient survey responses from patient-centered outcomes research. Together, these sources capture diverse linguistic styles, care contexts, and social environments. The annotated corpus contains 1137 messages, including 757 patient-authored and 380 provider-authored messages. In total, the dataset includes 46,038 word tokens, with an average message length of 40.5 words and a standard deviation of 32.8 words. Message length varies widely, ranging from brief clarifications with few words to long narrative descriptions, with the longest message containing 261 words, reflecting realistic properties of patient-generated text and posing meaningful challenges for structured extraction (Table 7).

**Table 7:** Demographic distribution across data sources

	YNHH	TXACC Woven	TXACC Bethesda	Survey	Total
Sex (Male)	81 (38%)	29 (48%)	71 (47%)	38 (26%)	219 (38%)
Sex (Female)	132 (62%)	32 (52%)	79 (53%)	109 (74%)	352 (62%)
Race (White)	152 (71%)	8 (13%)	20 (13%)	109 (74%)	289 (51%)
Race (Black)	35 (16%)	6 (10%)	15 (10%)	8 (5%)	64 (11%)
Race (Asian)	11 (5%)	4 (7%)	9 (6%)	19 (13%)	43 (8%)
Race (Other)	15 (7%)	43 (70%)	106 (71%)	11 (8%)	175 (31%)
Ethnicity (Hispanic)	30 (14%)	24 (39%)	60 (40%)	8 (5%)	122 (21%)

Percentages are calculated within each data source.

Although PVminer focuses on patient-generated text, provider-authored messages are intentionally included to preserve conversational context and enable models to distinguish intent and structure across message sources, reflecting real-world deployment conditions. Across all sources, the dataset includes message threads from 571 unique patients. Data from charitable clinics contribute linguistic, cultural, and socioeconomic diversity that complements messages from a large academic medical center, while survey responses broaden expression beyond clinical messaging systems. Using multiple data sources reduces institution-specific bias and improves generalization across heterogeneous settings. Training and testing splits are constructed at the message level using iterative stratification^58^ to preserve label coverage under the hierarchical, multi-label annotation schema. Each message is an individual, self-contained text authored at a distinct point in time. Message-level splitting reflects the intended deployment setting, in which the system processes new messages from patients whose earlier texts may have already been observed. Moreover, the PVminer task requires content-grounded extraction of Code, Sub-code, and Span triples, where correct predictions depend on the semantic content of each message rather than patient-specific writing patterns. Because messages from the same patient may appear in both partitions, this design may introduce patient-level information leakage and potentially overestimate performance. The resulting distribution of Code–Sub-code labels across training and testing splits is provided in Table S5.

Table 8 compares PVminer with representative biomedical NLP benchmarks across four properties essential for patient voice extraction. PVminer is the only benchmark that jointly supports relational and socioemotional content, bidirectional interaction, multi-label annotation, and alignment with secure messaging data. These properties introduce distinct modeling challenges: overlapping social signals require multi-label structured extraction, mixed-author message streams demand direction-aware label assignment, and informal patient-authored language necessitates robust Span grounding under linguistic variability.

**Table 8:** Comparison between PVminer and representative biomedical NLP benchmarks

Benchmark	Domain	Focus	Data Source	Rel./Socio- Emo.	Bidir. Interac.	Multi- Label	Secure Msg.
CBLUE^77^	Chinese biomedical	NER, RE, classification	Clinical text and dialogs	×	×	×	×
MedDG^78^	Chinese medical dialog	Diagnosis, symptom inquiry	Simulated dialogs, semi-structured dialogs	×	×	×	×
ReMeDi^79^	English medical dialog	Medication intent, treatment reasoning	Clinical conversations (non-SM)	△	△	×	×
MediTOD^80^	English history-taking	Symptom elicitation, clinical reasoning	Simulated structured dialogs	×	×	×	×
BLURB^81^	Biomedical NLP	NER, RE, QA, summarization	Biomedical literature	×	×	×	×
**PVminer(Ours)**	U.S. secure messaging	relational behaviors, adherence cues	De-identified SM from patient portals	*✓*	*✓*	*✓*	*✓*

*✓* indicates strong support, × indicates no support, and △ indicates partial or limited support. Benchmarks are compared along four properties relevant to patient voice extraction from patient-generated text. They are Relational/Socio-Emotional, Bidirectional Interaction, Supports Multi-Label Coding, and Tailored for Secure Messaging.

**Fig. 1:**
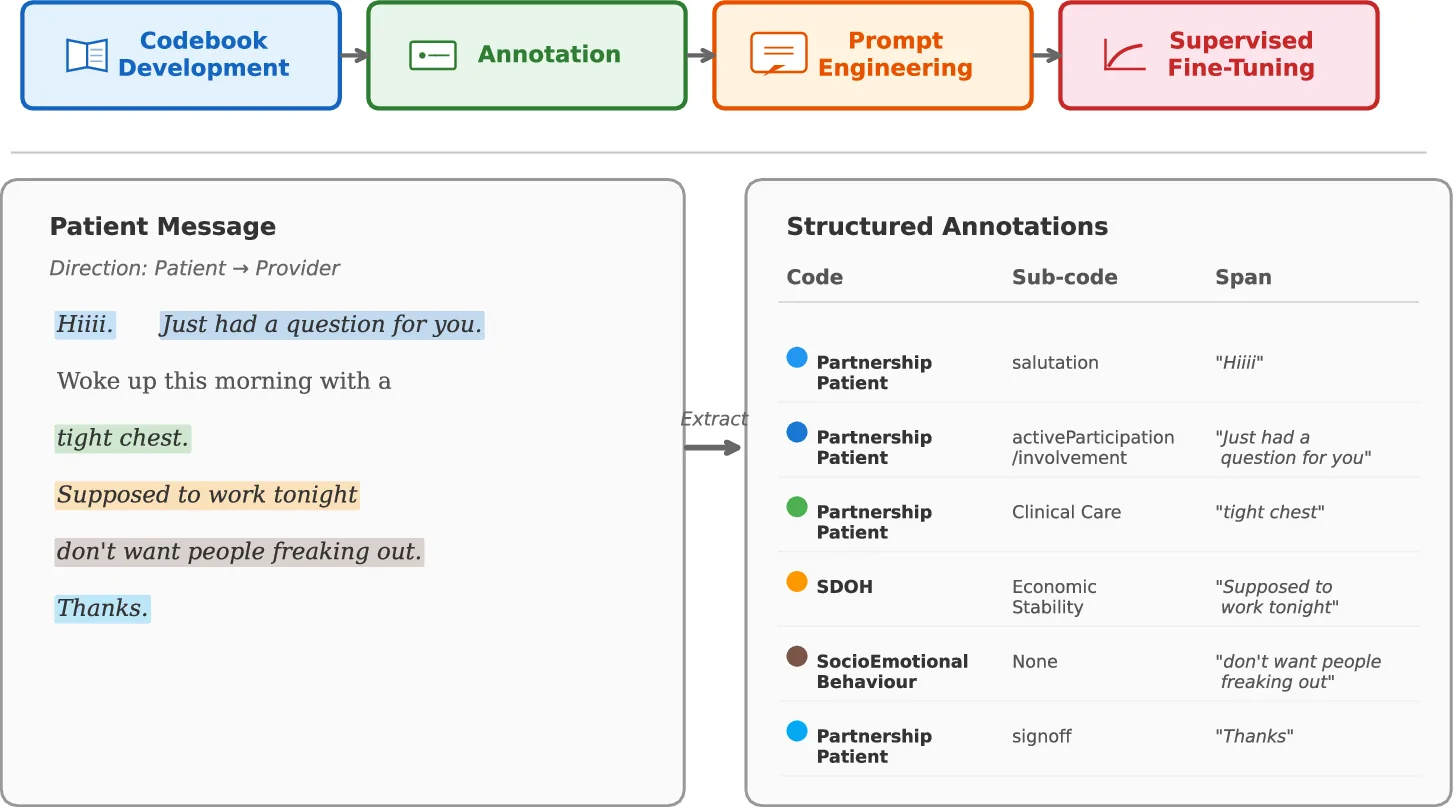
Overview of the PVminer framework. **Top**: the four-stage pipeline from codebook development through supervised fine-tuning. **Bottom**: a worked example illustrating the structured extraction task. Given a patient-generated message with direction metadata, the model extracts a set of (Code, Sub-code, Span) triples, where each Span is grounded verbatim in the input text. This example demonstrates the multi-label nature of the task: a single message yields six annotations spanning three distinct Codes, including partnership signals, a social determinant of health (employment concern), and a socioemotional expression.

We developed an annotation schema and a codebook to support structured extraction of patient voice from patient-generated text. The codebook captures socially and clinically meaningful expressions commonly found in secure messages and survey responses, while remaining compatible with schema-constrained modeling. All messages were annotated by domain experts in health communication and informatics using the eHOST platform^59^, following an iterative protocol with regular collective reviewing, ambiguity handling, and label definitions updating, which is shown in Fig. 1.

**Fig. 2:**
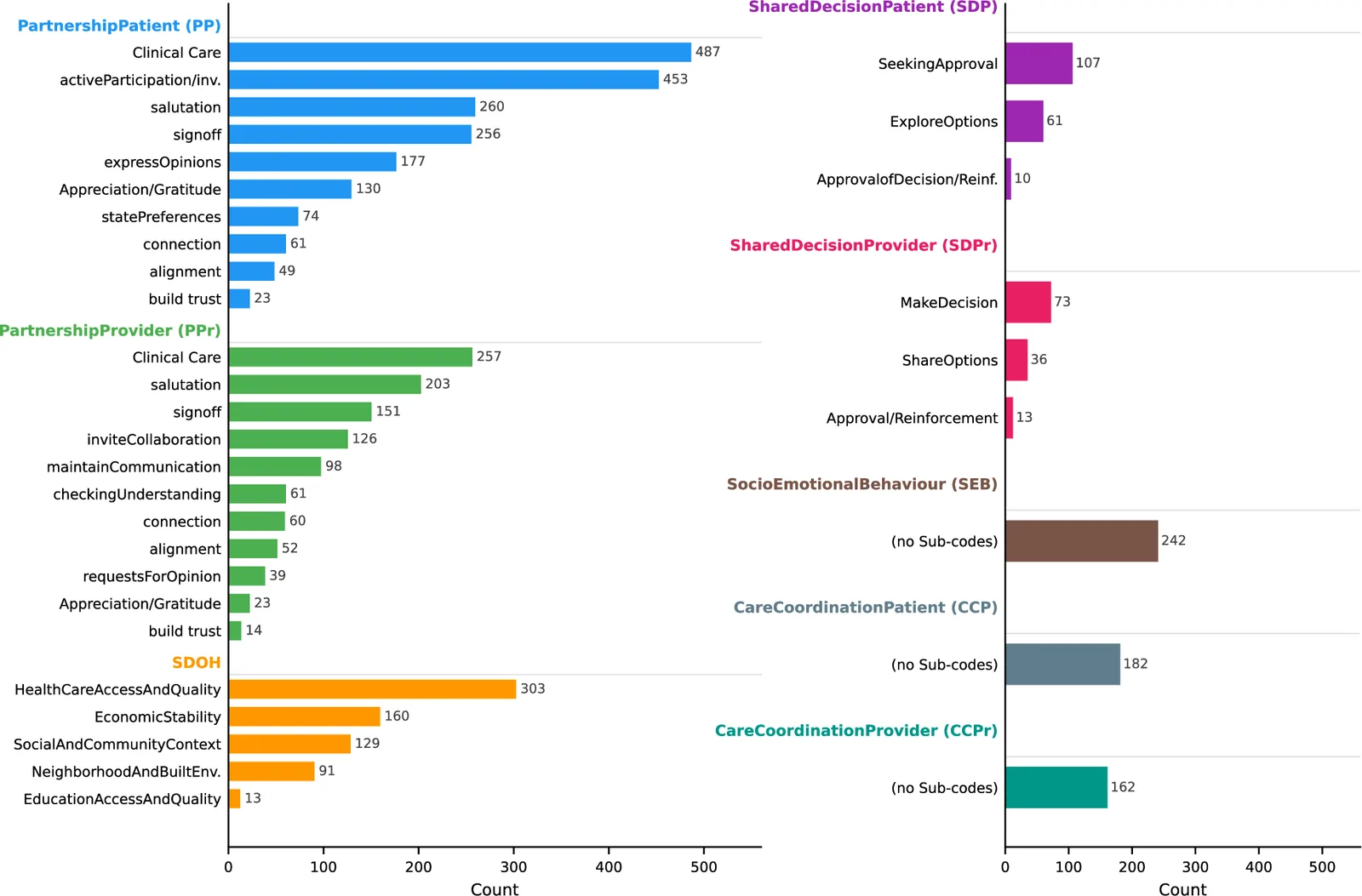
Distribution of Code–Sub-code pairs in the annotated dataset, grouped by Code. A small number of categories dominate the distribution, while many clinically meaningful Sub-codes occur in the long tail, posing challenges for both prompt-based and supervised extraction. The distribution across training and testing splits is provided in Supplementary Information.

The final annotation schema represents patient voice using a two-level hierarchical structure. Each annotation consists of a Code capturing a high-level communicative or social function, a Sub-code specifying finer-grained intent or context, and a Span grounding the label in the original text. This design allows multiple overlapping annotations per message and supports fine-grained, Span-level evaluation. Figure 2 shows that Code and Sub-code combinations are highly imbalanced, with a small number of frequent categories and a long tail of rare but semantically important cases. This reflects real-world patient-generated language and poses meaningful challenges for both prompt-based and supervised models. The schema comprises eight major Codes with associated Sub-codes; full definitions and examples are provided in Supplementary Information.

### Prompt engineering

To provide a benchmark, we establish a baseline prompt within the PVminer framework for evaluating large language models as structured annotators. Unlike open-ended generation tasks, PVminer requires outputs to be parseable, label-consistent, and grounded in exact text Spans. Prompt design therefore focuses on reliability and constraint satisfaction rather than linguistic fluency. For a patient-generated message *s* and a message-direction indicator *d*, models are prompted to produce schema-constrained outputs consisting of one or more Code, Sub-code, and Span tuples. *Baseline prompt* is used to characterize task difficulty and motivate subsequent prompt engineering and supervised adaptation. The baseline prompt is described as Supplementary Information.

To improve upon the baseline, we propose an *engineered prompt* that targets dominant failure modes observed in structured extraction: format drift (non-parseable or verbose outputs), semantic confusion between closely related labels, errors arising from implicit assumptions about speaker role, and Span boundary noise (incomplete or hallucinated Spans). The engineered prompt addresses these failures through integrated components, each motivated by established prompting principles and tailored to the constraints of token-critical structured generation. Our proposed engineered prompt is provided in Supplementary Information, which improves the baseline prompt through the following techniques:

#### Schema contracting

The prompt defines an explicit output contract in JSON format with hard constraints: Spans must be copied verbatim from the input, Code–Sub-code pairs must be valid under the predefined schema, and all applicable tuples must be returned. This reduces format drift and frames generation as a constrained decision problem rather than open-ended text completion^60,61^.

#### Direction-aware control logic

A major source of systematic error is confusion between patient-oriented and provider-oriented label variants (e.g., PartnershipPatient vs. PartnershipProvider). The prompt exposes message direction (TO_PAT_YN) as an explicit control variable with direction-conditioned rules that map semantic intent to the correct label family depending on speaker role^52,62^.

#### Chain-of-thought decomposition

Rather than relying on single-step label prediction, the prompt includes a 4-step reasoning routine: (1) context and direction analysis, (2) phrase decomposition and code matching, (3) span extraction and verification, and (4) cross-validation with loop-back conditions. This procedural scaffold induces deliberate decomposition and reduces brittle one-shot label guessing, following established chain-of-thought principles^63,64^.

#### Self-validation

The prompt concludes with a quality gate that instructs the model to internally audit its candidate output against concrete failure conditions, which include invalid label pairings, span invention, partial coverage, and disambiguation rule violations, before giving the final JSON. Unlike multi-turn self-refinement frameworks^65,66^, this operates within a single-query, single-response regime, functioning as an intra-generation verifier.

#### Expert-derived disambiguation rules

A dedicated disambiguation block addresses empirically observed label confusions (e.g., salutation vs. signoff, Appreciation/Gratitude vs. signoff, requestsForOpinion vs. inviteCollaboration). These rules were curated from ambiguity patterns encountered during expert annotation and serve an analogous function to an annotation manual^67,68^.

#### Role priming and performance targets

The prompt anchors the model in a domain-relevant persona and states explicit accuracy targets for Code, Sub-code, and Span prediction. These act as behavioral priors that bias toward conservative, defensible decisions^52,69^.

Collectively, the engineered prompt combines schema contracting, direction-aware control, chain-of-thought decomposition, single-turn self-validation, expert-derived disambiguation, and role priming into an integrated framework for token-critical structured generation. This design substantially improves schema validity and reduces common annotation errors (section “Baseline and engineered prompt performance on the PVminer benchmark”) and serves as the foundation for supervised fine-tuning in the subsequent section.

### PVminerLLM—supervised fine-tuned LLMs

We developed PVminerLLM by applying supervised fine-tuning to specialize instruction-tuned language models for the PVminer task, with the goal of improving reliability in structured extraction under strict schema constraints. Each training instance consists of a patient-generated message *s*, a message-direction indicator *d* ∈ {*Y*, *N*}, and a gold annotation set ${\mathcal{A}}$ containing one or more structured tuples of the form {Code, Sub−code, Span}. During fine-tuning, the model learns to produce schema-valid outputs with accurate hierarchical label assignment and exact Span grounding. This training-based approach addresses reliability limitations observed in prompt-only inference. For training, each structured annotation set ${\mathcal{A}}$ is serialized into a JSON-formatted target string *a*. The conditioning query *q* is formed by combining task instructions with the instance-specific message content and the message-direction indicator. The model is then trained to generate the structured completion *a* conditioned on *q*, thereby learning to map patient-generated text to schema-conformant annotations.

Formally, the conditioning query *q* is constructed as

3 $$q={\mathcal{I}}\,\parallel \,\backslash {\rm{n}}\,\parallel \,s\,\parallel \,\backslash {\rm{n}}\,\parallel \,d,$$

where ${\mathcal{I}}$ denotes the task instruction template, *s* denotes the patient-generated message text, *d* denotes the message-direction indicator, and ∥ represents string or token concatenation. The serialized target completion is defined as

4 $$a={\rm{Serialize}}({\mathcal{A}}),$$

where Serialize(⋅) maps the structured annotation set into a schema-valid JSON string.

Let **w** = [*w*_1_, …, *w*_*L*_] denote the token sequence obtained by encoding the concatenation of the query *q* and the target completion *a*. A binary mask **m** ∈ {0, 1}^*L*^ is applied where *m*_*t*_ = 1 if token *w*_*t*_ belongs to the serialized annotation *a*, and *m*_*t*_ = 0 otherwise. Tokens corresponding to task instructions and input context are excluded from optimization to ensure that learning focuses exclusively on structured output generation.

The supervised fine-tuning objective is defined as

5 $${{\mathcal{J}}}_{\sup }(\phi )=-{{\mathbb{E}}}_{(q,{\mathcal{A}}) \sim {\mathcal{D}}}\left[\frac{1}{\mathop{\sum }\nolimits_{t = 1}^{L}{m}_{t}}\mathop{\sum }\limits_{t=1}^{L}{m}_{t}\log {P}_{\phi }({w}_{t}| {w}_{ < t})\right],$$

where *P*_*ϕ*_ denotes the language model parameterized by *ϕ*. This masked likelihood objective prevents the model from memorizing task instructions and instead allocates learning capacity to producing valid Code and Sub-code combinations and character-exact Span boundaries^70^.

We implement supervised fine-tuning using parameter-efficient adapters with QLoRA^71^, applying low-rank updates to attention projection layers while keeping base model parameters fixed. This approach enables efficient adaptation across models of different sizes while maintaining computational feasibility. The resulting fine-tuned models, referred to as PVminerLLM, provide a practical and scalable solution for structured extraction of patient voice from patient-generated text.

### Evaluation metrics

We evaluate model performance using metrics tailored to the structured and multi-component nature of the PVminer task. Because each message may contain multiple overlapping labels and evidence Spans, evaluation is performed separately for Code prediction, Sub-code prediction, and Span extraction, with all metrics computed in a multi-label setting.

Code prediction is evaluated as a multi-label classification problem over the predefined set of Codes. Let ${\hat{y}}_{i}^{{\rm{Code}}}$ denote the set of Codes predicted for instance *i*, and let ${y}_{i}^{{\rm{Code}}}$ denote the corresponding gold standard set. Precision, recall, and F1-score are computed as

6 $${{\rm{precision}}}_{{\rm{Code}}}=\frac{{\sum }_{i}\left\vert {\hat{y}}_{i}^{{\rm{Code}}}\cap {y}_{i}^{{\rm{Code}}}\right\vert }{{\sum }_{i}\left\vert {\hat{y}}_{i}^{{\rm{Code}}}\right\vert },$$

7 $${{\rm{recall}}}_{{\rm{Code}}}=\frac{{\sum }_{i}\left\vert {\hat{y}}_{i}^{{\rm{Code}}}\cap {y}_{i}^{{\rm{Code}}}\right\vert }{{\sum }_{i}\left\vert {y}_{i}^{{\rm{Code}}}\right\vert },$$

8 $${{\rm{F}}1}_{{\rm{Code}}}=\frac{2\times {{\rm{precision}}}_{{\rm{Code}}}\times {{\rm{recall}}}_{{\rm{Code}}}}{{{\rm{precision}}}_{{\rm{Code}}}+{{\rm{recall}}}_{{\rm{Code}}}}.$$

Sub-code prediction is also evaluated as a multi-label classification task, where each message may be associated with multiple Sub-codes. Let ${\hat{y}}_{i}^{{\rm{Sub}}}$ and ${y}_{i}^{{\rm{Sub}}}$ denote the predicted and gold Sub-code sets for instance *i*, respectively. We compute

9 $${{\rm{precision}}}_{{\rm{Sub}}}=\frac{{\sum }_{i}\left\vert {\hat{y}}_{i}^{{\rm{Sub}}}\cap {y}_{i}^{{\rm{Sub}}}\right\vert }{{\sum }_{i}\left\vert {\hat{y}}_{i}^{{\rm{Sub}}}\right\vert },$$

10 $${{\rm{recall}}}_{{\rm{Sub}}}=\frac{{\sum }_{i}\left\vert {\hat{y}}_{i}^{{\rm{Sub}}}\cap {y}_{i}^{{\rm{Sub}}}\right\vert }{{\sum }_{i}\left\vert {y}_{i}^{{\rm{Sub}}}\right\vert },$$

11 $${{\rm{F}}1}_{{\rm{Sub}}}=\frac{2\times {{\rm{precision}}}_{{\rm{Sub}}}\times {{\rm{recall}}}_{{\rm{Sub}}}}{{{\rm{precision}}}_{{\rm{Sub}}}+{{\rm{recall}}}_{{\rm{Sub}}}}.$$

Evidence Span extraction is assessed using a relaxed token-level matching strategy designed to account for boundary ambiguity in natural language annotation. For each example *i*, let ${{\mathcal{S}}}_{{\rm{pred}}}^{(i)}$ represent the set of predicted Spans and ${{\mathcal{S}}}_{{\rm{ref}}}^{(i)}$ represent the set of reference Spans. A predicted Span ${s}_{p}\in {{\mathcal{S}}}_{{\rm{pred}}}^{(i)}$ is considered a true positive if it aligns with at least one reference Span ${s}_{r}\in {{\mathcal{S}}}_{{\rm{ref}}}^{(i)}$ according to any of the following conditions: (i) the reference Span is entirely contained within the predicted Span; (ii) the predicted Span is entirely contained within the reference Span; (iii) the token-level Jaccard overlap between the two Spans is greater than or equal to 0.6. Conditions (i) and (ii) handle cases where one Span is a subset of the other and are independent of the overlap threshold. A sensitivity analysis varying the Jaccard threshold in condition (iii) is provided in Supplementary Information. Precision, recall, and F1-score for Span extraction are computed as

12 $${{\rm{precision}}}_{{\rm{Span}}}=\frac{| {\rm{TP}}| }{| {\rm{TP}}| +| {\rm{FP}}| },$$

13 $${{\rm{recall}}}_{{\rm{Span}}}=\frac{| {\rm{TP}}| }{| {\rm{TP}}| +| {\rm{FN}}| },$$

14 $${{\rm{F}}1}_{{\rm{Span}}}=\frac{2\times {{\rm{precision}}}_{{\rm{Span}}}\times {{\rm{recall}}}_{{\rm{Span}}}}{{{\rm{precision}}}_{{\rm{Span}}}+{{\rm{recall}}}_{{\rm{Span}}}}.$$

### Experimental setting

All experiments are conducted using the lm_eval framework^72,73^ with a vLLM backend. We evaluate instruction-tuned large language models across a range of sizes, including Llama3.3-70B-Instruct, Llama3.1-8B-Instruct, Llama3.2-3B-Instruct^74^, and Qwen2.5-1.5B-Instruct^75,76^.

Prompt-based evaluation is performed in zero-shot and few-shot settings. Zero-shot experiments use a maximum context length of 8096 tokens, while few-shot experiments increase the context length to accommodate in-context exemplars. Generation is constrained to schema-valid JSON outputs and terminates at a designated stop string (for example, JSON_END), with a maximum of 1024 generated tokens per instance. Each model’s official chat template is applied at inference time, and a strict output contract is enforced to prevent extraneous text. All results are collected from experiments at temperature 0.7 across 3 runs.

For supervised fine-tuning, we use parameter-efficient QLoRA adapters while keeping base model parameters frozen. Training uses a maximum input length of 8192 tokens with bfloat16 precision, and the 70B model additionally applies 4-bit weight quantization. Gradient checkpointing and gradient accumulation are enabled to support long-context training. Optimization is performed with AdamW and a linear warmup schedule using the HuggingFace Trainer. All training runs are conducted on two H200 GPUs with distributed data parallelism.

## Supplementary information

Supplementary Information

## Data Availability

The data analyzed in this study consist of de-identified patient–provider secure messages and associated annotations derived from clinical systems. Due to privacy, ethical, and institutional restrictions, these data are not publicly available. Access to the data may be considered upon reasonable request to the corresponding author and with appropriate institutional review board (IRB) approval, and data use agreements in place.

## Code availability

Code, evaluation scripts, and trained LLMs are available at https://github.com/Data-Mining-Lab-Yale/PVminerLLM.
