# Supplementary material for: PVminerLLM: structured extraction of patient voice from patient-generated text using large language models
Source: NPJ Digit Public Health. 2026 Oct 27;1(1):44. doi: 10.1038/s44482-026-00039-1 (PMC13713561; doi:10.1038/s44482-026-00039-1)
Supplement: Supplementary file 1 [file 44482_2026_39_MOESM1_ESM.pdf]

Supplementary Information

Sensitivity Study Over the Threshold for Span Results

A sensitivity analysis varying the Jaccard threshold is provided in Table S1. Because the casual matching fact of the metric, the result is relatively robust to the change of threshold.

Table S1 Span F1 (mean ± std over 3 runs) as a function of the Jaccard matching threshold.

	Qwen2.5-1.5B		Llama3.3-70B	
Threshold	SFT	2-shot	SFT	2-shot
0.60	82.63 ± 0.32	48.92 ± 2.23	88.24 ± 0.29	68.88 ± 0.47
0.65	82.51 ± 0.27	48.15 ± 2.35	88.08 ± 0.30	68.53 ± 0.61
0.70	82.28 ± 0.20	47.70 ± 2.43	87.91 ± 0.32	67.95 ± 0.72
0.75	82.16 ± 0.20	47.26 ± 2.50	87.83 ± 0.31	67.60 ± 0.64
0.80	82.04 ± 0.20	46.88 ± 2.68	87.69 ± 0.35	66.97 ± 0.70
0.85	82.04 ± 0.20	46.71 ± 2.65	87.65 ± 0.37	66.72 ± 0.74
0.90	82.04 ± 0.20	46.68 ± 2.64	87.65 ± 0.37	66.72 ± 0.74
0.95	82.04 ± 0.20	46.68 ± 2.64	87.65 ± 0.37	66.72 ± 0.74

Qualitative Case Analyses

We present three representative test-set examples comparing two-shot and SFT predictions (70B model). Each case highlights a distinct failure mode at both the Code and Sub-code level. Correct Code–Sub-code pairs are marked with ✓ and incorrect with ×. For brevity, Codes are abbreviated: PP = PartnershipPatient, PPr = PartnershipProvider, SDP = SharedDecisionPatient, SDPr = SharedDecisionProvider, SEB = SocioEmotionalBehaviour.

Case 1: Incomplete extraction on a dense multi-label message.

"Hiiii. Just had a question for you. Woke up this morning with a tight chest and I have a dry cough when I take a deep breath. My back and neck are sore. I don't have a fever (98.6) o2 sat is 97-98. Supposed to work tonight don't want people freaking out. Call if you can. Thanks."

This patient message contains 6 unique Code–Sub-code pairs spanning clinical symptoms, an employment-related SDOH concern, and a socioemotional expression (Table S2). Two-shot extracts only 2 correct pairs: it confuses the Sub-code *activeParticipation/involvement* with *expressOpinions*, merges the SDOH (EconomicStability) and socioemotional signals into a single *statePreferences* label, and misses the Clinical Care Spans entirely. PVminerLLM identifies all 6 pairs with correct Sub-codes, separately extracting each clinical symptom, the employment concern, and the socioemotional content.

Table S2 Case 1: Dense multi-label patient message (6 gold pairs).

	Code	Sub-code	Span
Two-shot (2/6 correct pairs)			
✓	PP	salutation	"Hiiii"
×	PP	expressOpinions	"Just had a question..."
		[gold: activeParticipation/involvement]	
×	PP	statePreferences	"Supposed to work tonight..."
		[gold: SDOH/EconomicStability + SEB/None]	
×	SDP	SeekingApproval	"Call if you can"
		[gold: PP/activeParticipation]	
✓	PP	signoff	"Thanks"
SFT (6/6 correct pairs)			
✓	PP	salutation	"Hiiii"
✓	PP	activeParticipation	"Just had a question..."
✓	PP	Clinical Care	"Woke up..." (6 Spans)
✓	SDOH	EconomicStability	"Supposed to work tonight"
✓	SEB	None	"don't want people freaking out"
✓	PP	signoff	"Thanks"

Case 2: Direction confusion between patient and provider roles.

> *"So as of right now I have an appointment in the new office MM/DD/YYYY at 8 am. Then MM/DD/YYYY I start the medicine I inject? When is the actually produce going to be done and in what hospital? Is it a out patient procedure or do I stay for a few hours or a night? Just making sure I know all this."*

Despite the message direction indicating a patient-authored message, two-shot assigns exclusively Provider-side Codes and Sub-codes: all five predictions use SharedDecisionProvider/ShareOptions or PartnershipProvider/checkingUnderstanding, yielding 0 correct pairs (Table S3). This illustrates the direction confusion failure mode. PVminerLLM correctly identifies Patient-side Codes and Sub-codes, achieving 4 out of 5 correct pairs; the missed pair is SDOH/NeighborhoodAndBuiltEnvironment ("in the new office"), a subtle environmental signal.

Table S3 Case 2: Direction confusion on a patient message (5 gold pairs).

	Code	Sub-code	Span
Two-shot (0/5 correct pairs)			
×	SDPr	ShareOptions [gold: PP/Clinical Care]	"I have an appointment..."
×	SDPr	ShareOptions [gold: PP/Clinical Care]	"I start the medicine..."
×	SDPr	ShareOptions [gold: PP/activeParticipation]	"When is the actually..."
×	SDPr	ShareOptions [gold: SDP/ExploreOptions]	"Is it a out patient..."
×	PPr	checkingUnderstanding [gold: PP/activeParticipation]	"Just making sure..."
SFT (4/5 correct pairs)			
✓	PP	Clinical Care	"I have an appointment..." (2 Spans)
✓	PP	activeParticipation	"When is the actually..." (3 Spans)
✓	SDOH	HealthCareAccess	"in what hospital"
✓	SDP	ExploreOptions	"or do I stay..."
×	missed: SDOH/NeighborhoodAndBuiltEnv. ("in the new office")		

Table S4 Case 3: Missed SDOH signals (7 gold pairs).

	Code	Sub-code	Span
Two-shot (1/7 correct pairs)			
✓	SDP	SeekingApproval	"Is it possible to get..."
×	PP	expressOpinions [gold: SDOH/NeighborhoodAndBuiltEnv.]	"I won't be able to drive..."
×	PP	Appreciation/Gratitude [gold: PP/activeParticipation + PP/signoff]	"I just wanted to ask thank you"
SFT (7/7 correct pairs)			
✓	SDOH	NeighborhoodAndBuiltEnv.	"Ice on roads"
✓	SDP	SeekingApproval	"Is it possible to get"
✓	PP	Clinical Care	"my Adderall refilled early"
✓	SDOH	NeighborhoodAndBuiltEnv.	"bad weather"
✓	SDOH	NeighborhoodAndBuiltEnv.	"I won't be able to drive..."
✓	PP	activeParticipation	"I just wanted to ask"
✓	PP	signoff	"thank you"

Case 3: Missed social determinant signals.

"Ice on roads MM/DD/YYYY Is it possible to get my Adderall refilled early because of the bad weather on MM/DD/YYYY I won't be able to drive in it I just wanted to ask thank you Person1 XXX-XXX-XXXX"

This message contains three distinct NeighborhoodAndBuiltEnvironment Sub-code signals related to weather and transportation barriers (Table S4). Two-shot captures the medication request (SeekingApproval) but misses all three SDOH signals, misclassifying the transportation barrier ("I won't be able to drive") as PP/expressOpinions instead of SDOH/NeighborhoodAndBuiltEnvironment, and assigning PP/Appreciation/Gratitude to "thank you" instead of the correct Sub-code PP/signoff. PVminerLLM identifies all three SDOH references with the correct Sub-code and correctly distinguishes signoff from Appreciation/Gratitude.

Instruction Prompt Design

Prompt 1 - Baseline Prompt

```
You are a patient-centered communication analyst tasked with identifying and classifying how
patients and clinicians incorporate patient-centered communication (PCC) elements in secure
messaging.

Your goal is to extract multiple Code-Sub-code pairs from the current sentence and identify
specific spans corresponding to each pair. This task requires careful, step-by-step reasoning
to ensure accurate multi-label classification, with additional consideration of contextual
information from surrounding sentences.

## Follow these steps systematically and step-by-step Instructions:
1. Understand the Input Sentence:
1.1 Analyze the message to establish the full context.
1.2 Note:
1.3 Carefully read and analyze every word in the message to determine its context and identify
all relevant communication elements.
2. Identify Relevant Codes:
2.1 Match parts of the message to one or more Codes based on the intent and content described
in the definitions below.
2.3 Acknowledge that a message may involve multiple Codes.
3. Determine Sub-codes for Each Code:
3.1 For each identified Code, assign the appropriate Sub-code(s) that further specify the
meaning.
3.2 Use definitions of Sub-codes to ensure accuracy and consistency.
3.4 Important: Ensure that the Sub-code you select belongs to the Sub-code list under the
identified Code. If it doesn't, reconsider whether the Code or Sub-code selection is correct.
4. Pair Codes with Sub-codes:
4.1 Form unique Code-Sub-code pairs for the message. These pairs should fully describe the
meaning of the message.
4.2 If multiple Codes exist in the same message, their Sub-codes will differ.
5. Highlight Evidence for Each Pair:
5.1 Extract minimal, specific spans of text from the message that support each identified
Code-Sub-code pair.
5.2 Note: The extracted minimum span should be a core phrase in the message instead of the
entire sentence.

The following content provides definitions for Codes and Sub-Codes.

## Code and Definitions:

(Full list omitted here for brevity).

FORMAT (Code WITH Sub-codes):
CODE_NAME: <one-sentence operational definition>.
|- SUBCODE_1: <one-sentence operational definition>.
|- SUBCODE_2: <one-sentence operational definition>.
|- SUBCODE_K: <one-sentence operational definition>.

FORMAT (Code WITHOUT Sub-codes):
CODE_NAME: <one-sentence operational definition>.
```

```
|- None: No sub-codes are defined for this Code.

Ensure your reasoning is step-by-step to capture all relevant Code-Sub-code pairs and their
corresponding spans accurately. Remember, the Sub-code must belong to the list of Sub-codes
under the identified Code.

IMPORTANT: Output your final result without any explanation and reasoning, you must output the
JSON format like "results": ["Code": "<Identified Code>_1", "Sub-code": "<Identified
Sub-code>_1", "Span": "<Extracted span>_1",...,"Code": "<Identified Code>_n", "Sub-code":
"<Identified Sub-code>_n", "Span": "<Extracted span>_n"]
```

Prompt 2 - Engineered Prompt

- XML Structuring
- Chain-of-Thought (4-step reasoning)
- Self-Validation (quality gate)
- Decision Logic (direction-aware)
- Disambiguation Rules
- Performance Targets

```
<role>
Expert patient-centered communication analyst with >95% accuracy in medical message
multi-label classification.
</role>

<performance_target>
CRITICAL REQUIREMENTS:
- Code Accuracy: >95%
- Sub-code Accuracy: >95%
- Span Accuracy: >98% (character-perfect)

Every annotation must be defensible and verification-validated.
</performance_target>

<task>
Extract Code, Sub-code, and Span triples from medical secure messages.

INPUT:
- Context (the message text)
- Message Direction (TO_PAT_YN)

OUTPUT:
- JSON list of {Code, Sub-code, Span} objects

CONSTRAINT:
- MULTI-LABEL task (one message may contain multiple valid triples)
</task>

<message_direction>
CRITICAL: Message direction determines Code selection.

- TO_PAT_YN = "Y": Provider speaking TO patient
- TO_PAT_YN = "N": Patient speaking TO provider

USE CASES:
- Provider to patient: Use PartnershipProvider, SharedDecisionProvider,
  CareCoordinationProvider when applicable
- Patient to provider: Use PartnershipPatient, SharedDecisionPatient,
  CareCoordinationPatient when applicable
- SDOH and SocioEmotionalBehaviour apply regardless of direction
```

```
</message_direction>

<critical_rules>
RULE VIOLATIONS RESULT IN ANNOTATION FAILURE

1. Span Source:
   - Extract Spans ONLY from the provided message text
   - Context is for understanding only
   - Never invent, paraphrase, or infer Spans

2. Code and Sub-code Validity:
   - Every Sub-code MUST be valid for its Code
   - If a pairing is illogical or invalid, loop back and re-select
```

```
3. Span Exactness:
   - Copy EXACT text from the message
   - Preserve punctuation, capitalization, and spacing
   - No paraphrasing

4. Multi-label Requirement:
   - Identify ALL relevant Code and Sub-code pairs in the message
</critical_rules>

<reasoning_process>
Follow this 4-step verification process:

STEP 1: CONTEXT AND DIRECTION ANALYSIS
- Read the full message carefully
- Determine message direction using TO_PAT_YN
- Understand speaker intent and conversational goal

STEP 2: PHRASE DECOMPOSITION AND CODE MATCHING
- Break the message into semantic units (phrases or clauses)
- For each phrase, identify intent:
  * SDOH
  * PartnershipProvider or PartnershipPatient
  * SharedDecisionProvider or SharedDecisionPatient
  * CareCoordinationProvider or CareCoordinationPatient
  * SocioEmotionalBehaviour
- Use TO_PAT_YN to select Provider vs Patient variants
- Match each phrase to the correct Code definition
- Verify Code and Sub-code pairing is logical and valid

STEP 3: SPAN EXTRACTION AND VERIFICATION
- Extract the MINIMUM complete supporting phrase
- Spans must come ONLY from the message text
- Verify character-level exactness
- If the Span does not exist exactly, reject the annotation

STEP 4: CROSS-VALIDATION (MOST IMPORTANT)
Verification priority:
1. Best semantic match confirmed (if not, loop back to Step 2)
2. Sub-code valid for Code
3. Span is exact and present in message
4. All relevant phrases analyzed
5. Disambiguation rules applied correctly
6. High-confidence annotation defensible to experts
</reasoning_process>

<codes_definitions>
The following are authoritative ground-truth definitions. Names must match exactly.
(Full list omitted here for brevity).

FORMAT (Code WITH Sub-codes):
```

```
CODE_NAME: <one-sentence operational definition>.
|- SUBCODE_1: <one-sentence operational definition>.
|- SUBCODE_2: <one-sentence operational definition>.
|- SUBCODE_K: <one-sentence operational definition>.

FORMAT (Code WITHOUT Sub-codes):
CODE_NAME: <one-sentence operational definition>.
|- None: No sub-codes are defined for this Code.
</codes_definitions>

<disambiguation_rules>
Apply systematically to resolve ambiguity.

Salutation vs Signoff:
- Opening greetings indicate salutation
- Closing phrases indicate signoff
- Position determines classification
```

```
Appreciation/Gratitude vs Signoff:
- Simple closing thanks indicates signoff
- Specific appreciation indicates Appreciation/Gratitude

Provider vs Patient Codes:
- Use TO_PAT_YN strictly
- TO_PAT_YN = "Y" -> Provider codes
- TO_PAT_YN = "N" -> Patient codes

SharedDecision Codes:
- Use TO_PAT_YN to select Provider vs Patient variants

SDOH Sub-code Selection:
- EconomicStability: finances, income, food, housing
- EducationAccessAndQuality: education, literacy
- HealthCareAccessAndQuality: access to care, insurance, physical activity
- NeighborhoodAndBuiltEnvironment: housing, transportation, environment
- SocialAndCommunityContext: social support, isolation, discrimination

CareCoordination vs maintainCommunication:
- maintainCommunication: future updates only
- CareCoordination: concrete coordination with other providers

requestsForOpinion vs inviteCollaboration:
- requestsForOpinion: asks patient views
- inviteCollaboration: invites joint participation

SocioEmotionalBehaviour:
- Emotional support, reassurance, empathy, politeness
- Only Sub-code "None" is valid
</disambiguation_rules>

<output_format>
Return JSON with a "results" array:

{
  "results": [
    {
      "Code": "exact Code name",
      "Sub-code": "exact Sub-code name",
      "Span": "EXACT text from message"
    }
  ]
}

If no annotations apply:
{"results": []}
```

```
</output_format>

<quality_gate>
MANDATORY verification before submission:

1. JSON is parseable
2. All Sub-codes valid for Codes
3. All Spans are exact and present in message
4. Best semantic match verified
5. All disambiguation rules applied
6. High confidence suitable for expert review

Accuracy is paramount. Quality over speed.
</quality_gate>

INPUT:
TO_PAT_YN: N (Patient speaking to provider)
```

```
Context:
Dr. Person1 I need my prescription sent to the pharmacy for my flecainide acetate
100 mg tablets twice a day the pharmacist has try requesting it no success and I
don't have any pills. Person2
```

Detailed CodeBook

The annotation schema consists of eight major Codes, each representing a distinct communicative or social construct within the patient voice. Each major code has a corresponding set of Sub-codes that capture more specific communicative intents. Below are concise summaries of these key categories:

1. Social Determinants of Health (SDOH). Refers to the process of sharing and seeking knowledge about the social, economic, and environmental factors that significantly influence an individual's health and well-being. These include economic stability, access and quality to education, access and quality of health care, neighborhood and built environment, and social and community context. *Subcodes:*

- **Economic Stability:** Financial security and resources to afford healthcare, housing, food, and other necessities.
- **Education Access and Quality:** Provide information on the availability and effectiveness of educational opportunities, the quality of education, including resources, teaching standards, and curriculum.
- **Access and quality to healthcare:** The ability to obtain the necessary health services and the effectiveness and standard of care that patients receive.
- **Neighborhood and Built Environment:** Physical and social surroundings such as housing, transportation, safety, and environmental quality.
- **Social and Community Context:** Social and community context refers to relationships, interactions, and conditions within the environments where people live, work, and interact, and it significantly influences health outcomes.

2. Partnership from the Patient Side. Patient partnership involves establishing and strengthening the alliance between patients and healthcare providers through active participation, open communication, and mutual respect. *Subcodes:*

- **Active Participation/Involvement:** Patient is active in providing information to aid diagnosis and problem solving, priorities for treatment or management, asking questions and/or contributing to the identification of management approaches.
- **Express Opinions:** Patients actively share their thoughts, concerns about their care and treatment and provide feedback on their experiences with healthcare services.
- **Signoff:** Courteous closure that marks the completion of a message.
- **State Preferences:** Individual values, desires, and priorities of the patient regarding their healthcare.
- **Alignment:** Refers to establishment of a meaningful, trust-based relationship between the patient and healthcare provider.
- **Appreciation/Gratitude:** appreciation and gratitude that are expressed when patients acknowledge the care and support they receive.
- **Connection:** Information that is not directly related to the medical issue being discussed, strengthening the relationship among between the patient and provider.
- **Salutation:** Greeting or addressing the provider by name or title.
- **Clinical Care:** Refers to the patient's experiences, perceptions, and self-reported expressions concerning symptoms, diagnoses, treatments, or medical procedures they receive or seek.
- **Build Trust:** Fostering a sense of confidence and reliability, wherein the patient feels assured that the provider is acting in their best interest.

3. Partnership from the Provider Side. Refers to fostering a collaborative and equitable relationship between healthcare providers and patients, involving the equalization of status and ensuring that patients feel valued and empowered. *Subcodes:*

- **Invite Collaboration:** Inviting patients to participate in decisions related to their condition through informed consent, treatment planning, and self-management.
- **Requests for Opinion:** actively seeking patients' perspectives and preferences on treatment options, care plans, and health-related decisions.
- **Checking Understanding/Clarification:** Confirming that the patient fully understands the information being communicated, including key concepts related to their condition, treatment options, costs, and care plan.
- **Appreciation/Gratitude:** Refers to the expression of appreciation and gratitude by healthcare providers when acknowledging patients' engagement, cooperation, and participation in their care.
- **Signoff:** Courteous ending signaling completion of communication.

- **Maintain Communication:** It involves keeping patients informed and engaged throughout the care process by clearly communicating that additional information or updates will be provided at a later time, ensuring patients are aware of what to expect.
- **Alignment:** Confirming or seeking confirmation that the patient and provider share a mutual understanding and perspective on a given issue.
- **Connection:** Comments or information unrelated to the medical issue that serve to strengthen the patient–provider relationship and foster rapport.
- **Salutation:** Refers to the act of greeting or respectfully addressing the patient.
- **Clinical Care:** Refers to the provider's planning, coordination, and delivery of diagnoses, treatments, and medical procedures tailored to the patient's clinical needs.
- **Build Trust:** Involves fostering a relationship with the patient characterized by mutual respect, transparency, and reliable communication, which is essential for effective healthcare delivery.

4. Shared Decision-Making from the Patient Side. Involves patients actively engaging in treatment discussions, asking questions, and providing informed approval of decisions that align with their values and goals, while fostering confidence in the chosen course of action. *Subcodes:*

- **Explore Options:** Indicators of a patient's interest in learning about all options include asking detailed questions, expressing curiosity about alternatives, seeking clarification, discussing how options align with personal goals, and requesting additional resources for further information.
- **Seeking Approval:** Refers to the patient asking for or seeking permission from the healthcare provider.
- **Approval of Decision:** Affirmation refers to the patient agreeing with a chosen plan or decision after understanding the available options and their implications.

5. Shared Decision-Making from the Provider Side. Refers to statements or questions that create an opportunity for a two-way exchange of information regarding treatment options, evaluation of decisions, or consideration of alternatives. *Subcodes:*

- **Share Options:** Refers to the process of identifying, presenting, and deliberating the available options for a specific decision or problem, encompassing a discussion of their associated risks, benefits, and potential outcomes.
- **Make Decision:** Refers to the collaborative process in which the provider and patient jointly select a course of action after considering all available options, associated risks and benefits, and the patient's personal preferences.
- **Approval of Decision/Reinforcement:** Refers to reinforcing a decision by providing positive feedback or validating the patient's involvement in the decision-making process.

6. Socioemotional Behavior. Refers to responding to patients' emotional cues by validating their feelings, offering empathy, reassurance, and encouragement, acknowledging errors transparently, and providing positive reinforcement, reward, or approval. No Sub-codes are defined.
7. Care Coordination Provider. Refers to the deliberate organization and integration of healthcare services by the provider to ensure that patient care is delivered effectively, efficiently, and in a timely manner. No Sub-codes are defined.
8. Care Coordination Patient. Represents the patient's active engagement in communicating and coordinating care among various healthcare providers and services to ensure continuity and effectiveness. No Sub-codes are defined.

Label Distribution Across Training and Testing Splits

The distribution of Code–Sub-code labels across training and testing splits is shown in Table S5. Sub-codes are grouped by parent Code and sorted by total frequency in descending order.

Table S5 Distribution of Code–Sub-code labels across training and testing splits, counted at the message level. Sub-codes are grouped by parent Code and sorted by total frequency in descending order.

Code	Sub-code	Train	Test	Total
PartnershipPatient	Clinical Care	386	101	487
	activeParticipation/involvement	362	91	453
	salutation	208	52	260
	signoff	205	51	256
	expressOpinions	139	38	177
	Appreciation/Gratitude	104	26	130
	statePreferences	60	14	74
	connection	47	14	61
	alignment	37	12	49
	build trust	19	4	23
PartnershipProvider	Clinical Care	201	56	257
	salutation	158	45	203
	signoff	117	34	151
	inviteCollaboration	96	30	126
	maintainCommunication	77	21	98
	checkingUnderstanding/clarification	47	14	61
	connection	48	12	60
	alignment	40	12	52
	requestsForOpinion	28	11	39
	Appreciation/Gratitude	18	5	23
	build trust	10	4	14
SDOH	HealthCareAccessAndQuality	239	64	303
	EconomicStability	121	39	160
	SocialAndCommunityContext	101	28	129
	NeighborhoodAndBuiltEnvironment	69	22	91
	EducationAccessAndQuality	9	4	13
SharedDecisionPatient	SeekingApproval	83	24	107
	ExploreOptions	49	12	61
	ApprovalofDecision/Reinforcement	8	2	10
SharedDecisionProvider	MakeDecision	53	20	73
	ShareOptions	28	8	36
	Approval/Reinforcement	9	4	13
SocioEmotionalBehaviour	None	178	64	242
CareCoordinationPatient	None	143	39	182
CareCoordinationProvider	None	127	35	162
